# Improving predicted protein loop structure ranking using a Pareto-optimality consensus method

**DOI:** 10.1186/1472-6807-10-22

**Published:** 2010-07-20

**Authors:** Yaohang Li, Ionel Rata, See-wing Chiu, Eric Jakobsson

**Affiliations:** 1 Department of Computer Science, Old Dominion University, Norfolk, VA 23529, USA; 2 Department of Molecular and Integrative Physiology, Department of Biochemistry, UIUC Programs in Biophysics, Neuroscience, and Bioengineering, National Center for Supercomputing Applications, and Beckman Institute, University of Illinois, Urbana, IL 61801, USA

## Abstract

**Background:**

Accurate protein loop structure models are important to understand functions of many proteins. Identifying the native or near-native models by distinguishing them from the misfolded ones is a critical step in protein loop structure prediction.

**Results:**

We have developed a Pareto Optimal Consensus (POC) method, which is a consensus model ranking approach to integrate multiple knowledge- or physics-based scoring functions. The procedure of identifying the models of best quality in a model set includes: 1) identifying the models at the Pareto optimal front with respect to a set of scoring functions, and 2) ranking them based on the fuzzy dominance relationship to the rest of the models. We apply the POC method to a large number of decoy sets for loops of 4- to 12-residue in length using a functional space composed of several carefully-selected scoring functions: Rosetta, DOPE, DDFIRE, OPLS-AA, and a triplet backbone dihedral potential developed in our lab. Our computational results show that the sets of Pareto-optimal decoys, which are typically composed of ~20% or less of the overall decoys in a set, have a good coverage of the best or near-best decoys in more than 99% of the loop targets. Compared to the individual scoring function yielding best selection accuracy in the decoy sets, the POC method yields 23%, 37%, and 64% less false positives in distinguishing the native conformation, indentifying a near-native model (RMSD < 0.5A from the native) as top-ranked, and selecting at least one near-native model in the top-5-ranked models, respectively. Similar effectiveness of the POC method is also found in the decoy sets from membrane protein loops. Furthermore, the POC method outperforms the other popularly-used consensus strategies in model ranking, such as rank-by-number, rank-by-rank, rank-by-vote, and regression-based methods.

**Conclusions:**

By integrating multiple knowledge- and physics-based scoring functions based on Pareto optimality and fuzzy dominance, the POC method is effective in distinguishing the best loop models from the other ones within a loop model set.

## Background

Protein loop structure modeling is important in structural biology for its wide applications, including determining the surface loop regions in homology modeling [1], defining segments in NMR spectroscopy experiments [2], designing antibodies [3], and modeling ion channels [4,5]. Typically, the protein loop structure modeling procedure involves the following steps [6,7]. First of all, the structural conformation space is sampled to produce a large ensemble of backbone models satisfying certain conditions such as loop closure, clash-free, and low score (energy). Secondly, clustering algorithms are applied to select representative models from these backbone models. Thirdly, side chains are added to the representative models to build all-atom models and their structures are further optimized by score minimization. Finally, the models are assessed and the "best" ones will be selected as the predicted conformations.

In many loop modeling methods [6-13], sample loop conformations are constructed by dihedral angle buildup or fragment library search [14]. Recently, Mandell et al. [15] developed a kinematic closure approach, which can construct loop conformations within a 1A resolution. Nevertheless, scoring functions used to guide loop modeling vary widely. Rohl et al. [8] optimized the Rosetta score using fragment buildup. Fiser et al. [9] used a hybrid scoring function by summing up CHARMM force field terms and statistically derived terms. Xiang et al. [10] developed a combined energy function with force-field energy and RMSD (Root Mean Square Deviation) dependent terms. They also developed the concept of "colony energy" that has been used by Fogolari and Tosatto [16] as well, for considering the loop entropy (an important component in flexible loops) as part of the total free energy. Olson et al. [17] used a multiscale approach based on physical potentials. An efficient grid-based force field has been employed by Cui et al. [18]. Jacobson et al. [6], Zhu et al. [7], Rapp and Friesner [11], de Bakker et al. [12], Felts et al. [13], and Rapp et al. [19] employed physics-based energy schemes with various solvent models. Soto et al. [20] found that using the statistical potential DFIRE [21] as a filter prior to all-atom physics-based energy minimization can improve prediction accuracy and reduce computation time. DFIRE has previously proven to be successful by itself for loop selection [21]. All these methods have led to recent significant progress in generating high-resolution loop models and several loop prediction servers are now available (see [22], for example).

In practice, the value of computer-generated protein loop models in biological research relies critically on their accuracy. While efficiently sampling the protein loop conformation space to produce sufficient number of low-energy models to cover conformations with good structures remains a challenging issue, another critical problem is the insensitivity of the existing protein scoring functions. These scoring functions are developed to estimate the energy of the protein molecule. The insensitivity of the scoring functions leads to difficulty in distinguishing the native or native-like conformations from the erroneous models, and thus restricts the loop structure prediction accuracy. Therefore, selecting the highest quality loop models from a number of other models is a critical step in solving the protein loop structure prediction problem.

The scoring functions play a significant role in protein structure assessment and selection. Although a number of scoring functions are currently available for protein loop model evaluation, there is no generally reliable one that can always distinguish the native or near native models. Every existing scoring function has its own pros and cons. Recently, the strategy of using multiple scoring functions to estimate the quality of models and improve selection was proposed in protein folding and protein-ligand docking [23-27]. Multiple, carefully selected scoring functions are integrated and selection improvements can be achieved by tolerating the insensitivity and deficiency of every individual scoring function. Thus, the multiple scoring functions method can usually lead to a better performance than an individual scoring function.

Similar to structure prediction in an overall protein, the scoring functions that have been used in loop modeling can be categorized into knowledge-based [8,21,28-30] and physics-based [13,31-35]. The knowledge-based scoring functions are typically derived from protein structural databases such as the PDB and thus incorporate empirical criteria to distinguish the native structure from the misfolds. By contrast, the physics-based scoring functions are developed based on first principle concepts, where electrostatic, Van der Waals, hydrogen bonds, solvation, and covalent interactions are taken into account.

**Figure 1:**
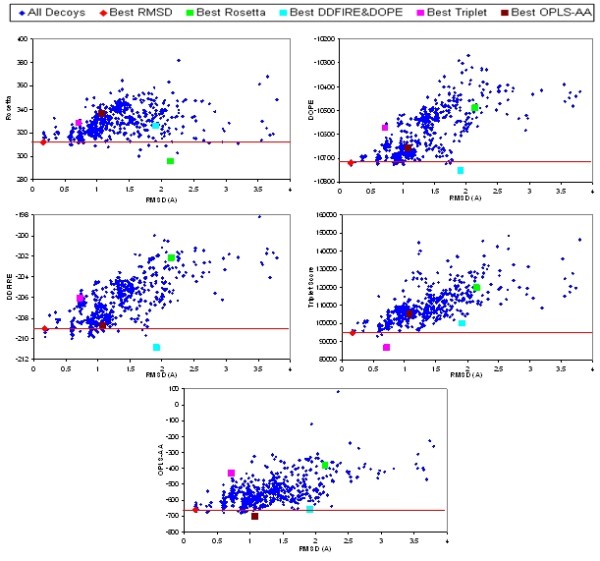
**RMSD-Score Plot of **1onc**(70:78) Decoy Set in Various Scoring Functions**

There are problems in theoretical justification of both the physics- and knowledge-based scoring functions for protein structure modeling. Ideally, a physics-based scoring function would be evaluated with quantum mechanics, in which case the score could reflect the true energy. In computation practice, quantum mechanics is wildly intractable due to the size of protein molecule. As a compromise, the physics-based scoring functions (force fields) are developed mainly based on classical physics to approximate the true energy of a protein molecule. On the other hand, the knowledge-based functions derive their rules from the existing experimental structure data, typically by applying the inverse Boltzmann law. However, because compared to the unknown structures, the known structures are in an extremely small fraction, the data used to develop knowledge-based functions are potentially undersampled [36,37]. Moreover, studies have shown that inter-residue interactions may not be considered as independent factors [38,39], which violates the assumption of inverse Boltzmann law. In consequence, all these aspects led to inaccuracy or insensitivity factors in the existing scoring functions for protein loop modeling, as is true in overall protein structure modeling. That is, in practice, the native conformation usually does not exhibit the lowest score when it is put among the models generated by the computer simulation program [40]. Moreover, in the low score regions, a conformation with a relatively higher score may in fact be a more reasonable structure than the one with a lower score. The score-RMSD plots in Figure 1 show that in the decoy set of 1onc(70:78), the best model (0.17A RMSD from the native) never yields the lowest score in DFIRE [21], triplet backbone dihedral potential [28], OPLS-AA/SGB [31,32], Rosetta [41], or DOPE [42], which strongly indicates insensitivity in each individual scoring function.

In this paper, we present a Pareto Optimality Consensus (POC) method based on the Pareto optimality [43] and fuzzy dominance theory [44] to take advantage of multiple scoring functions for ranking protein loop models. The rationale is to identify the models at the Pareto optimal front of the function space of a set of carefully selected scoring functions and then to rank them based on the fuzzy dominance relationship relative to the other models. For protein loop structure ranking, we employ five knowledge- or physics-based scoring (energy) functions: DFIRE [21], our triplet backbone dihedral potential [28], OPLS-AA/SGB [31,32], all-atom Rosetta [41], and DOPE [42]. All of these scoring functions have shown efficiency in loop modeling in the literature [6-8,21,28]. We apply our approach to the loop decoy sets generated by Jacobson et al. [6]. The loops in Jacobson's decoy sets are regarded as "difficult" targets [21,35]. There are frequent Pro and Gly occurrences in these loops. Cys are treated separately in both reduced and oxidized forms to take the formation of disulfide bridges into account. The loop positions are random to make possible encountering of all sorts of situations. Jacobson's decoy sets have been frequently used as a benchmark for loop prediction and effectiveness of scoring functions [20,21,35]. The original loop decoy sets include targets whose native protein structures have certain exceptional features such as high or low pH values when crystallized, explicit interactions between the target loops and heteroatoms, and low resolution crystal structures in target loop regions with large measured B-factors [6]. Jacobson et al. also provide a filtered list of decoy sets by eliminating targets with the above exceptional features. Since none of the scoring functions we used makes assumptions of these exceptional features, we only consider the filtered decoy sets in this paper. In addition to Jacobson's decoy sets, we apply our method to more recent decoy sets for 294 loops chosen from 44 chains in 38 membrane proteins [45]. We also compared the POC method with the hydrophobic potential of mean force (HPMF) approach for loop model selection as well as other multiple scoring functions ranking strategies [23], including Rank-by-Number, Rank-by-Rank, Rank-by-Vote, and regression-based methods.

## Methods

### The consensus Strategy

**Figure 2:**
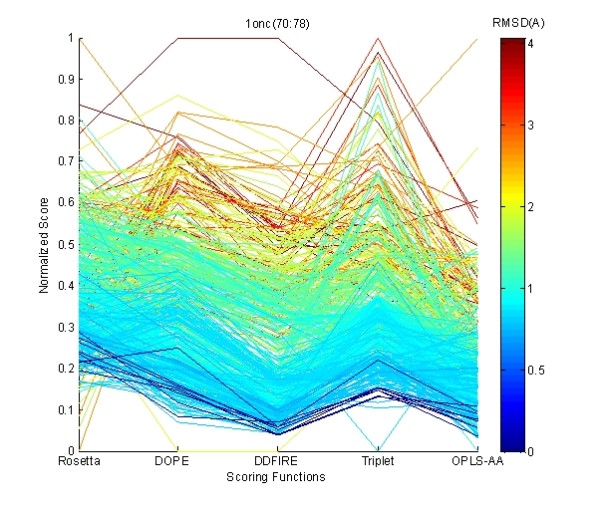
**Multiple Scoring Functions Coordinate Plot of Decoys in **1onc**(70:78) Decoy Set**

Although each scoring function may have certain insensitivity and inaccuracy, combining multiple, carefully selected scoring functions may effectively tolerate the deficiencies existent in the single scoring functions. For example, as shown in Figure 1, models yielding lowest score in one individual scoring function have higher scores than the best model in other scoring functions. However, the multiple coordinate plot in Figure 2 shows that the decoys commonly yielding low scores in all scoring functions are the best decoys in the 1onc(70:78) decoy set. As a result, efficiently integrating multiple good scoring functions may lead to resolution improvement in selecting the best decoys in the decoy set.

### The Pareto Optimality Consensus Method

The rationale of the POC method is to rank a model according to its Pareto-dominance relationship to the other models in the model set. The first step of the POC method is to identify models with Pareto-optimality. The definition of the Pareto-optimality [43] is based on the dominance relationship among models in the model set. A model *u *is said to dominate another model *v *(*u *≺ *v*) if both conditions i) and ii) are satisfied:

i) for each scoring function *f*_*i*_(.), *f*_*i*_(*u*) ≤ *f*_*i*_(*v*) holds for all *i*;

ii) there is at least one scoring function *f*_*j*_(.) where *f*_*j*_(*u*) <*f*_*j*_(*v*) is satisfied.

By definition, the models which are not dominated by any other models in the model set form the Pareto-optimal solution set. A Pareto-optimal model possesses certain optimality compared to the other ones in the model set.

**Figure 3:**
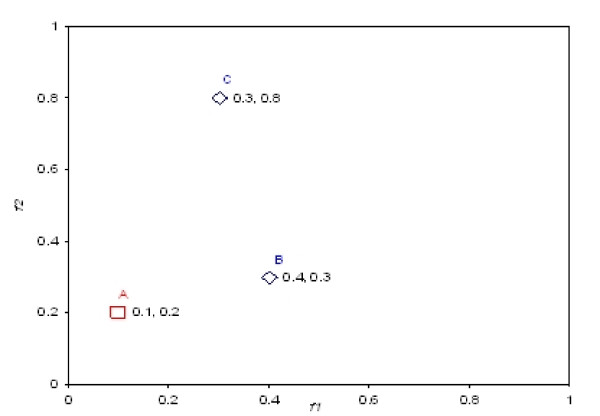
Example of Fuzzy Pareto Dominance

Once the Pareto-optimal models are identified, the next step in the POC method is to rank these models, so that the model that exhibits most optimality over other models in the model set will have the best rank. A simple solution, which is used in several evolutionary algorithms for multi-objective optimization [43], is to count the number of models in the model set that each Pareto-optimal model dominates. Then the Pareto-optimal model that dominates most other models is top-ranked. The major disadvantage of this simple solution is that it cannot accurately measure the significance of the dominance relationship between two decoys. Figure 3 shows an example of 3 models in a two-dimensional functional space, where A dominates both B and C while B and C do not dominate each other. The simple solution is not able to distinguish between the dominance relationships *A *≺ *B *and *A *≺ *C*, although A seems to have a "stronger" degree of dominance to C than to B.

To more accurately measure the dominance relationship, we adopt a fuzzy scheme [44] for model ranking. First of all, a function *g*(*f*_*i*_(*x*)): [min(*f*_*i*_(*x*)), max(*f*_*i*_(*x*))] → [0, 1] is used to normalize each scoring function *f*_*i*_(*x*). Then, a bounded division operation, ÷, is defined as

Finally, the fuzzy Pareto dominance relation between two models *u *and *v *can be written as follows: model *u *dominates model *v *by degree *μ*_*a *_where

for all normalized scoring functions *g*(*f*_*i*_(*.*)). Similarly, model *u *is dominated by model *v *by degree *μ*_*p *_where

for all normalized scoring functions *g*(*f*_*i*_(*.*)). In our current POC method, we use a linear membership function, min(*x*, *y*)/y, as suggested in [44], and the fuzzy scheme does not bias to any individual scoring functions.

For the example shown in Figure 3, *μ*_*a*_(A, C) = 1.0, *μ*_*p*_(A, C) = 0.083, *μ*_*a*_(A, B) = 1.0, and *μ*_*p*_(A, B) = 0.167. As a result, A shows a more significant dominance to C than to B in the fuzzy dominance scheme.

The ranking value for model *x*_*i*_, *r*(*x*_*i*_), is computed as

which will be used to rank the Pareto-optimal models. For ranking of the whole model set, we firstly identify the Pareto-optimal models and rank them according to fuzzy Pareto dominance relationship. Then, we remove the Pareto-optimal models, identify the Pareto-optimal models for the rest of the models, and assign ranks to them. The procedure is repeated until there are no more models left in the model set.

## Results

### Effectiveness of the Pareto Optimal Models

**Figure 4:**
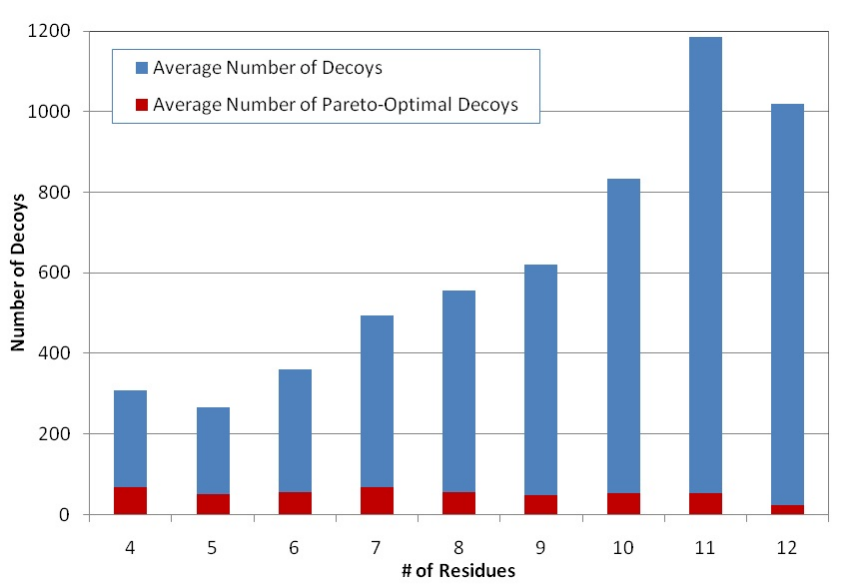
**Average number of decoys and average number of Pareto-optimal decoys for loop targets ranging from 4- to 12-residue in Jacobson's decoy sets**. Only a small fraction (3~22%) of the decoys of a loop target are Pareto-optimal decoys.

**Figure 5:**
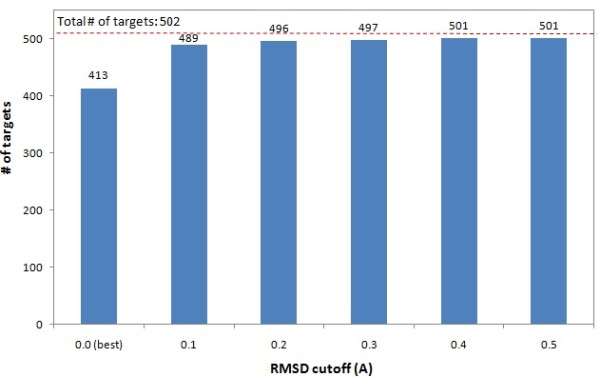
**Number of the targets whose Pareto-optimal decoys contain at least one decoy within certain RMSD cutoff from the best decoy**. The Pareto-optimal decoys can effectively cover the best decoy or one close to the best decoy in a target's decoy set.

**Figure 6:**
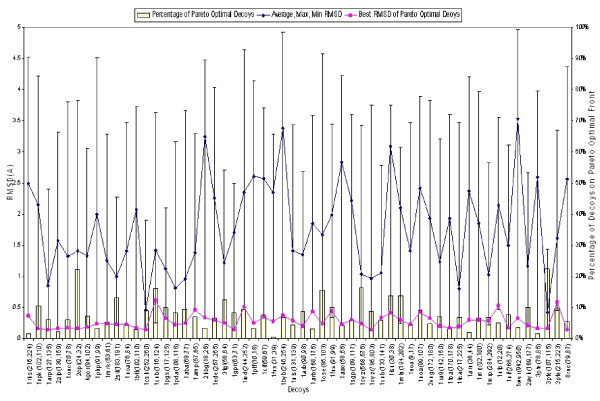
**Effectiveness of the Pareto optimal decoys**. The best decoy with minimum RMSD, or one very close to the best decoy (< 0.1A) are within the Pareto optimal decoys in 9-residue loop targets

Because in the POC method, selection and ranking are based on Pareto optimality, the quality of the Pareto-optimal models is critical. The Pareto-optimal models include not only those optimums in individual scoring functions, but also the non-dominated ones yielding certain optimality in the (linear or non-linear) combination of various scoring functions. In our computational experiment, five scoring functions, including Rosetta, DDFIRE, DOPE, triplet backbone dihedral, and OPLS-AA/SGB, are selected to form the function space. Figure 4 shows that the average number of the Pareto optimal decoys is around 20% or less of the total number of decoys in the Jacobson's decoy sets for 4- to 12-residue targets. As shown in Figure 5, the Pareto optimal decoys have efficient coverage of the best decoy or one close to the best decoy in a target's decoy set. In more than 82% of the loop targets, the Pareto-optimal decoys include the best decoy of the target, whereas in more than 97% of the loop targets, the Pareto-optimal decoys include decoys within 0.1A RMSD to the best one. Moreover, 501 out of 502 targets include decoys within 0.4A RMSD cutoff to the best decoy. Figure 6 shows the RMSD distribution of the decoys in the sets corresponding to the 9-residue loop targets as well as the coverage of the Parento optimal decoys. One can find that in most of the 9-residue targets, the very best decoy is in the Pareto-optimal decoy set, which typically contains only 5%~20% of the decoys from the original decoy set. This indicates that the selected scoring functions can efficiently identify a much smaller set of decoys that contains the best decoy or one very close to the best.

### Efficiency in Identifying Near-Native Structures

We applied the POC method to the decoy sets generated by Jacobson et al. The decoy set for each target contains very good models (MODEL 1 and MODEL 2) derived from the native structure by optimizing the OPLS-AA/SGB force field as well as other models generated by hierarchical comparative modeling [6].

**Figure 7:**
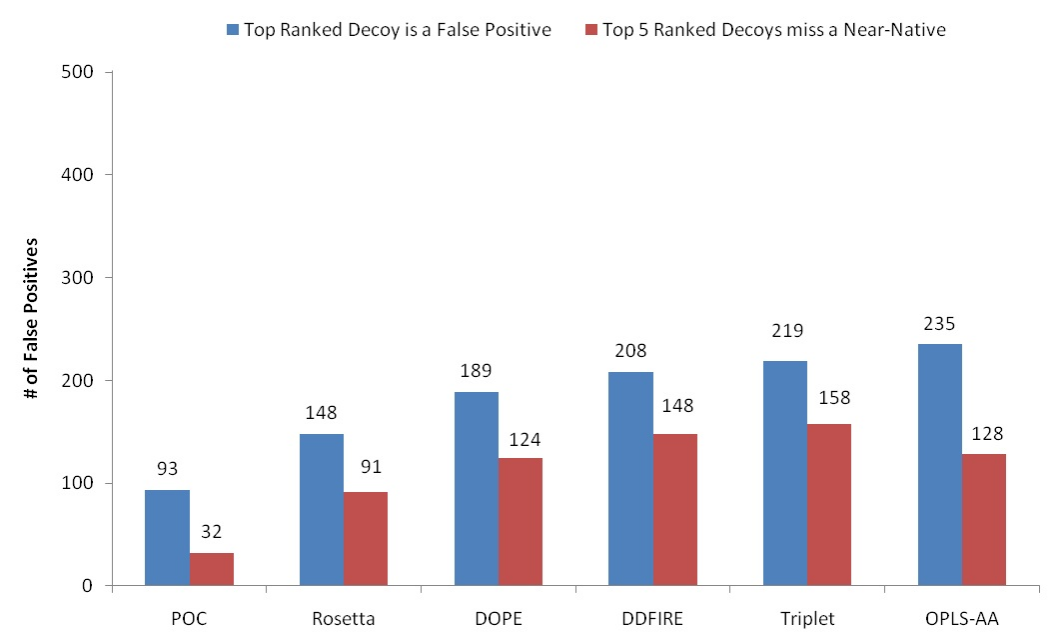
**Number of False Positives**. Number of cases where the top-ranked decoy is a false positive and the near-native structures are missed in the top-5-ranked decoys in 502 loop targets in the POC method and individual scoring functions

By considering a decoy with RMSD less than 0.5A as a near-native one, a false positive is a non-near-native decoy with a high rank. Figure 7 shows the overall percentages of the targets in which the top-ranked decoy is a false positive and the top-5-ranked decoys are all false positives in the 502 loop targets as scored by POC and the individual scoring functions. One can find that each individual scoring function we employed has rather high accuracy, yielding less than 50% false positive rate in ranking a near-native decoy as the top decoy. However, by integrating these scoring functions using fuzzy dominance, the POC method leads to 37.3% less false positives than the best individual scoring function in identifying the top-ranked decoy as a near-native. More significantly, the POC method has 64.6% less cases where the top-5-ranked decoys do not include a near-native model than those of the best individual scoring function.

**Figure 8:**
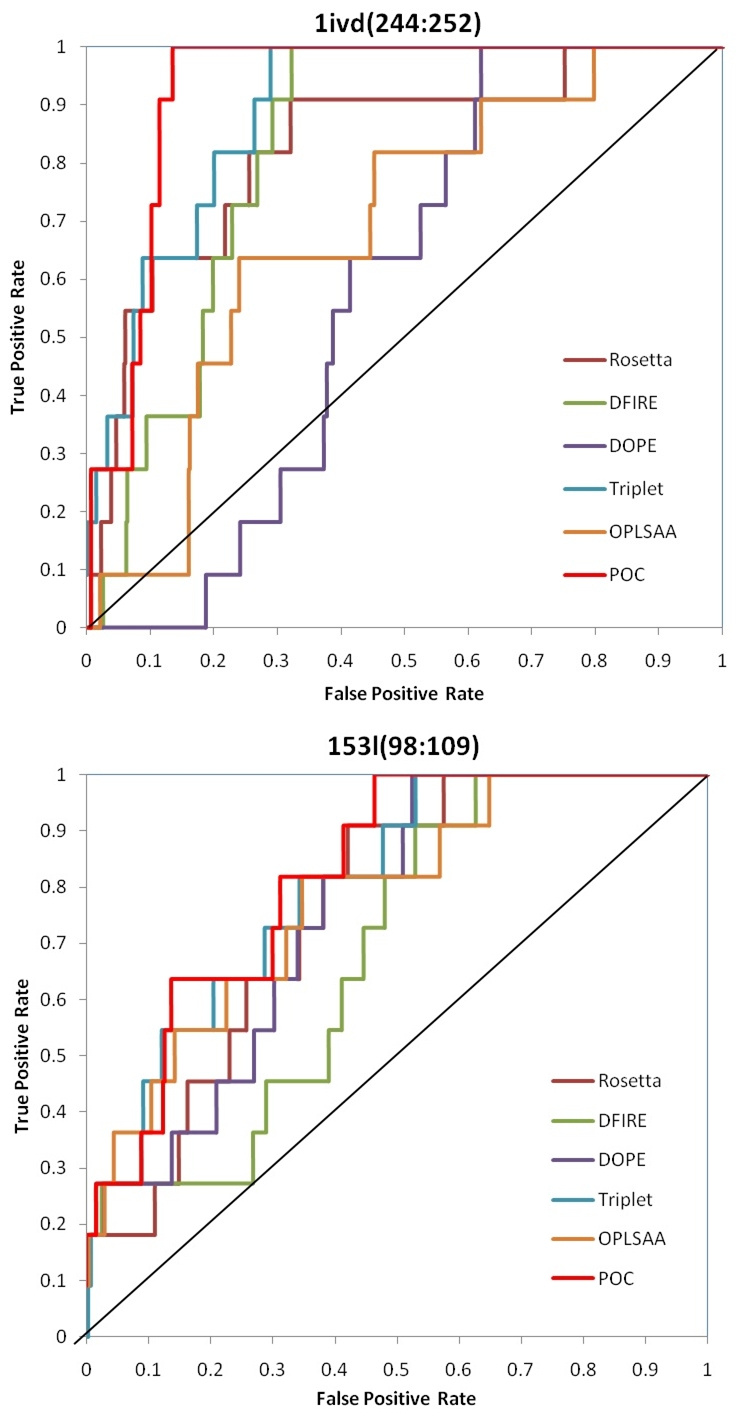
**ROC Curves for Decoys in **1ivd**(244:252) and 153 l(98:109)**. In these ROC curves, the true positives are the number of top-*N *ranked decoys with RMSD less than or equal to *r*, the false positives are the number of top-*N *ranked decoys with RMSD greater than *r*, the false negatives are the number of decoys with RMSD less than or equal to *r *but having rank greater than *N*, and the true negatives are the number of decoys with rank greater than *N *and RMSD greater than *r*. In our ROC plots, *r *is the 10^th ^best RMSD in a decoy set and *N *is the cutoff variable. The ROC curves generated by the POC method yield higher AUC values than those of the individual scoring functions.

We use the receiver operating characteristic (ROC) curves to evaluate the ranking performance of each individual scoring function as well as the POC method for each loop target, according to the method described in [46] for ranked data. ROC curves display the true positive rate versus the false positive rate. The area under the ROC curve (AUC) is determined from these ROC curves. An AUC of 1.0 indicates perfect ranking of the top *N *decoys whereas an AUC of 0.5 is representative of a random ranking. The higher an AUC value, the better the ranking performance. Figure 8 shows the ROC curves for evaluating the top-10-ranking of decoys in 1ivd(244:252) and 153 l(98:109). One can find that the POC method yields larger ROC AUC than individual scoring functions. Moreover, Table 1 shows the average ROC AUC values of individual scoring functions and POC in Jacobson's decoy sets and the membrane protein loop decoy sets, where Rosetta and DFIRE are the most effective individual scoring functions, respectively. POC yields even higher AUC value than Rosetta and DFIRE, as well as other scoring functions, in both cases. The OPLSAA score is not evaluated in membrane protein loop decoy sets because hydrogen atoms in the decoys are not available.

**Table 1:** Average ROC-AUC Comparison in Jacobson's Decoy Sets and the Membrane Protein Loop Decoy (MPD) Sets

	POC	Rosetta	DFIRE	DOPE	Triplet	OPLSAA
Jacobson	0.780920	0.752171	0.741472	0.737116	0.747701	0.608012

MPD	0.640534	0.592584	0.635511	0.612899	0.606396	N/A

**Figure 9:**
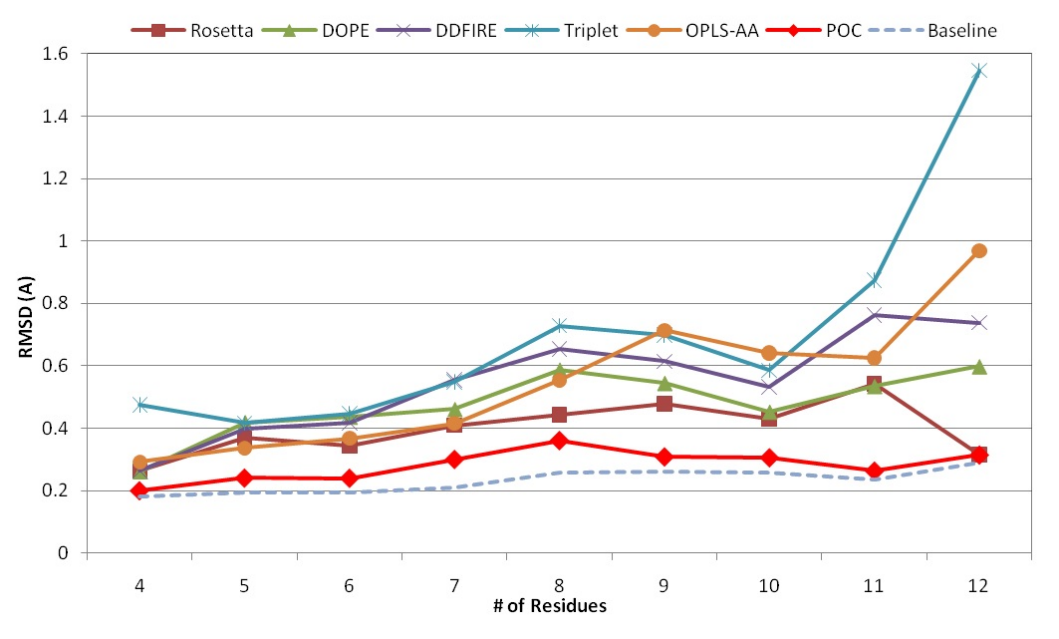
**Average RMSD of the best models selected from 5-top-ranked decoys in Jacobson's loop sets ranging from 4 to 12 residues**.

Figure 9 presents the average RMSDs of the best of the top-5-ranked decoys in POC method compared to the individual scoring functions in Jacobson's decoy sets for 4- through 12-residue loop targets. The POC method outperforms the individual scoring functions on 4- through 11-residue loop targets and is at least as good as the best individual scoring function (Rosetta) in 12-residue ones. The average RMSDs of the best of the top-5-ranked decoys selected by the POC method are rather close to the baseline formed by the average RMSD values of the best decoys in loop targets of various lengths. More interestingly, for each individual scoring function, there is strong correlation between the selected model's RMSD and the length of the loop target. By contrast, in the POC method, the dependence on the quality of the selected decoys with the length of the loop is hardly noticeable.

**Figure 10:**
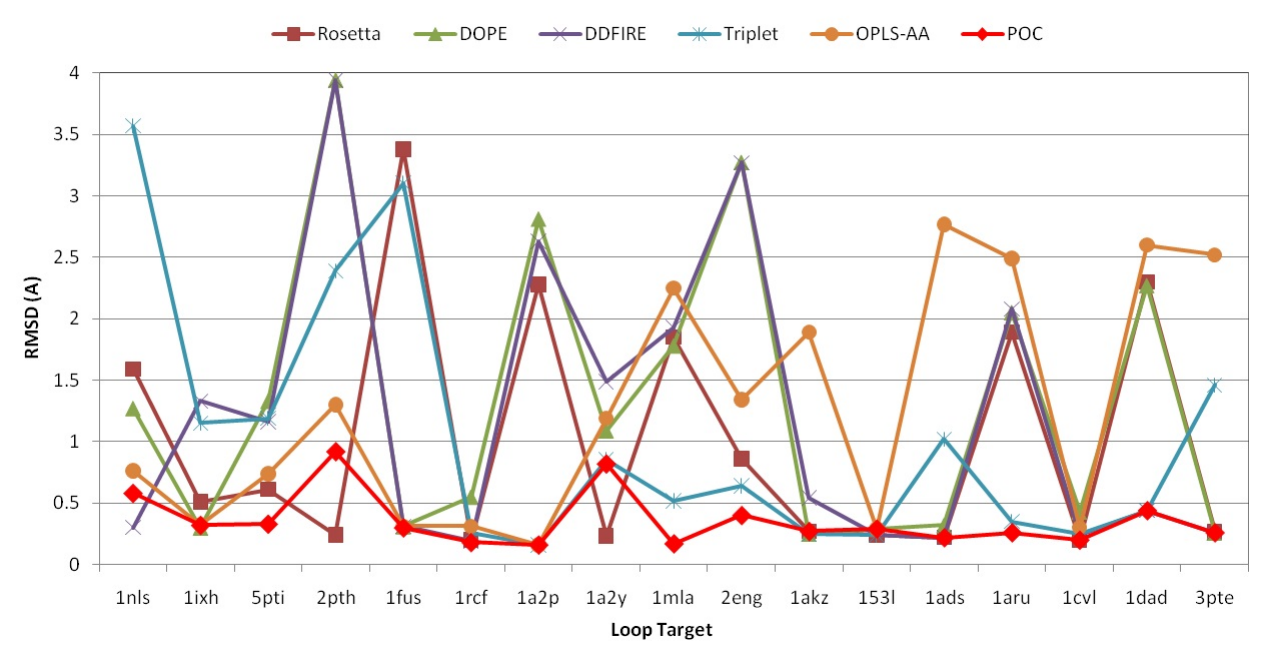
RMSD of the best-ranked decoy in 11-residue loop targets of Jacobson's decoy sets

Figure 10 shows the RMSD of the top-ranked decoy by the POC method as well as the individual scoring functions in 11-residue loop targets. One can notice that the individual scoring functions behave differently on various loop targets. There is no superior individual scoring function that can always select the native-like decoy. An individual scoring function may find the native-like decoys in some loop targets but miss the good ones in the other targets. By integrating these scoring functions, the POC method often coincides with the scoring function with correct decoy selection and rarely agrees with a scoring function pointing to an erroneous decoy. More interestingly, the top-ranked decoy in POC method is often better than all the top-ranked decoys in individual scoring functions, as shown for the loop targets 5pti(7:17), 1mla(9:19), 2eng(124:134), and 1aru(297:307).

**Figure 11:**
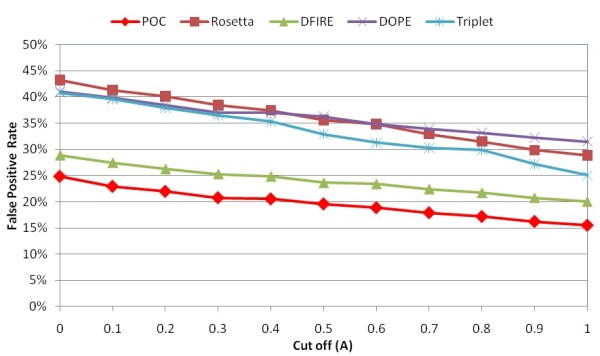
Comparison of false positive rates in POC and individual scoring functions using different RMSD cutoffs in membrane protein loop decoy sets

**Figure 12:**
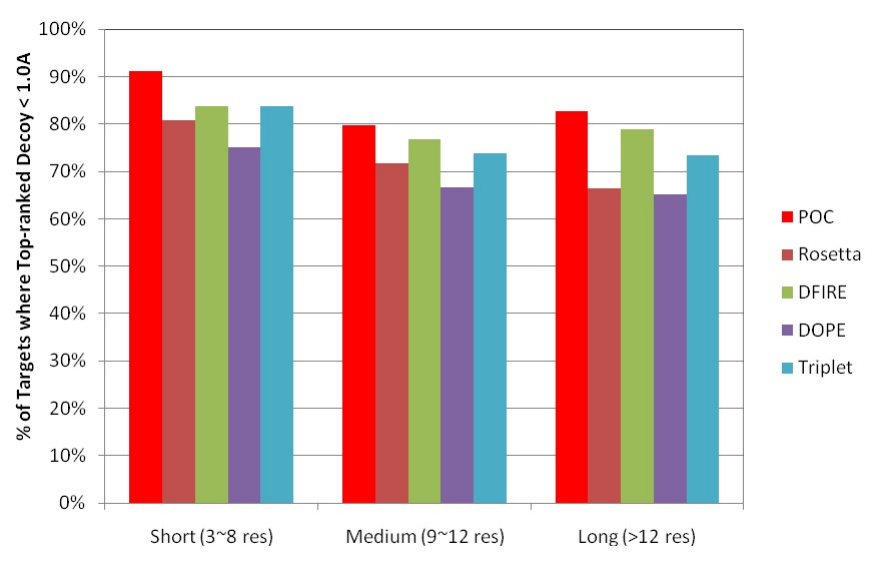
Percentage of targets in the membrane protein loop decoy sets where the top-ranked decoy is within 1.0A from the native

Respectively, Figures 11 and 12 show the false positive rates using different RMSD cutoffs and the percentage of targets with a top-ranked decoy within 1A RMSD from the native in the membrane protein loop decoy sets. One can find that DFIRE yields the best overall performance compared to the other individual scoring functions. Similar to our results in Jacobson's decoy sets, POC yields lower false positive selections than the best individual scoring function in the membrane protein loop decoy sets.

We also applied the POC method with the native structure mixed in the decoy sets generated by Jacobson et al [6]. Similar effectiveness of the POC method can be found in [Additional file 1].

## Discussion

### Comparison to Regression-based Consensus Method

**Figure 13:**
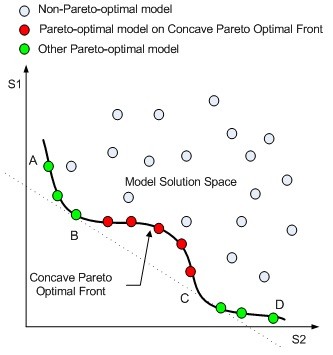
**Deficiency of Regression-based Consensus Method**. Pareto-optimal Models at the Concave Pareto Optimal Front Are Unreachable in a Regression-based Consensus Scoring Function

A popular approach to take advantage of multiple scoring functions is to build a consensus scoring function by combining the individual scores using linear regression [24]. However, the disadvantage of the regression-based consensus scoring function method is that it will overlook some models with certain optimality when the Pareto optimal front of the scoring function space is concave. Figure 13 shows a schematic illustration of a concave search space of two scoring functions *S*_*1 *_and *S*_*2*_. When a set of weights are determined by regression, a contour line is formed and the minimum solution of the consensus function corresponds to a model on the Pareto optimal front, which is the tangent point of the contour line and the model solution space. However, there exists no contour line that can produce a tangent point with the feasible solution space in the region BC in the Pareto optimal front. This is because before a tangent point is reached in BC, the contour line becomes a tangent at another point at AB or CD zones, which yields a lower overall consensus function value. In other words, models in the concave region BC will never be selected in a consensus scoring function method, although these models show certain Pareto-optimality relative to others in the model set. Some regions in the Pareto optimal front may still be unreachable even if nonlinear regression is used to combine various terms.

**Figure 14:**
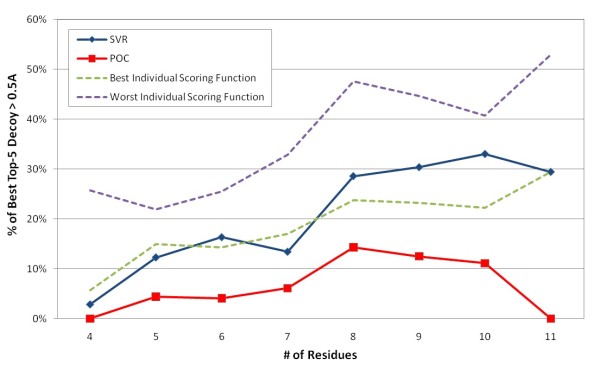
Selection performance comparison between POC and SVR in identifying the top-5 decoys in Jacobson's decoy sets

Figure 14 shows the performance comparison between a regression-based method using Support Vector Regression (SVR) [24] and POC in Jacobson's decoy sets. Linear SVR is regarded as a convex optimization method [47]. In the training process of the SVR method, for loops of a certain length, we randomly divided the loops into two roughly equal sets. We used one set as a training set and predicted the other set. Then we used the other set as the training set to predict the previous training set. For training and predicting, we used the libSVM library [48] with linear kernel and default parameters. One can find that SVR is close to or slightly outperforms the best individual scoring function in most cases. However, POC outperforms SVR in all lengths of targets in Jacobson's decoy sets.

Another major drawback of the regression-based consensus method is its dependence on the size, composition and generality of the training set used to derive the weights. Similar to the vote-based or rank-based consensus methods, POC does not require a training procedure. The selection and ranking solely depend on evaluation of the dominance relationship among the decoys.

### Comparison to Rank-by-Number, Rank-by-Rank, and Rank-by-Vote Methods

The vote-based consensus method is another strategy of multiple scoring functions selection method, which takes advantage of the observation that similar models voted by more scoring functions tend to be more accurate than those having fewer votes. However, the disadvantage of vote-based consensus methods is that it is very sensitive to the artificially-set vote threshold value [23,27]. Also, the vote-based consensus method has difficulties in situations when the scoring functions strongly disagree with each other. As a result, the vote-based consensus methods are usually inferior to the consensus score methods and are generally not recommended [23].

Table 2 shows the decoy selection comparison of the POC method and other consensus strategies [23], including rank-by-number, rank-by-rank, and rank-by-vote, in Jacobson's decoy sets of 502 loop targets. The rank-by-number is a straightforward consensus method, where all decoys are ranked according to the average normalized score values given by all scoring functions. The rank-by-rank strategy ranks the decoys according to their average ranks in each individual scoring function. In rank-by-vote strategy, a decoy will receive a vote by an individual scoring function if its score is the top *k*% (*k *= 2 in the results presented in Table 2) in the decoy set. Then, the decoys are ranked according to the number of votes each decoy received. Compared to the best individual scoring function, the rank-by-vote strategy has better selection accuracy in the top-ranked decoys and has similar performance in the top-5-ranked decoys. In agreement with [23], rank-by-number and rank-by-rank outperforms rank-by-vote in both top-ranked decoy and top-5-ranked decoys. The POC method yields more aggressive native-like decoy identification than rank-by-number and rank-by-rank, particularly in selecting the top-5-ranked decoys. This is due to the fact that the top-5-ranked decoys produced by the POC method have broader representation of the Pareto-optimal decoys than the other consensus strategies.

**Table 2:** Selection Accuracy Comparison of Various Consensus Strategies and Best Individual Scoring Function in Jacobson's Decoy Sets of 502 Loop Targets

	POC	Rank-by-Number	Rank-by-Rank	Rank-by-Vote	Best Individual Scoring Function
Top-ranked decoy < 0.5A	409	397	399	379	357

Best Top-5-ranked decoys < 0.5A	470	444	445	412	413

### Comparison to Another Selection Method

Lin and Head-Gordon recently presented a new physics-based energy function with an implicit solvent model, so-called HPMF [35], which has shown improved native-like loop discrimination capability from non-native decoys compared to DDFIRE and OPLS-AA/SGB, particularly in the long loop targets. Table 3 presents the average RMSD of the top-ranked decoys in loop targets with lengths ranging from 4 to 12 residues. The top-ranked decoys selected by POC method yield better average RMSD than those selected by HPMF in short, medium, and long loop targets.

**Table 3:** Selection Accuracy of the POC method compared to the HPMF Method

Loop Length	HPMF	POC
4	0.31A	0.27A

6	0.61A	0.34A

8	0.70A	0.53A

10	0.77A	0.49A

11	0.67A	0.39A

12	0.39A	0.32A

### Result Analysis

In this section, we analyze, from the biological perspective, the results obtained for several loop targets. These targets include 1fus(28:38), 1aac(16:20), and 1hbq(31:38).

**Figure 15:**
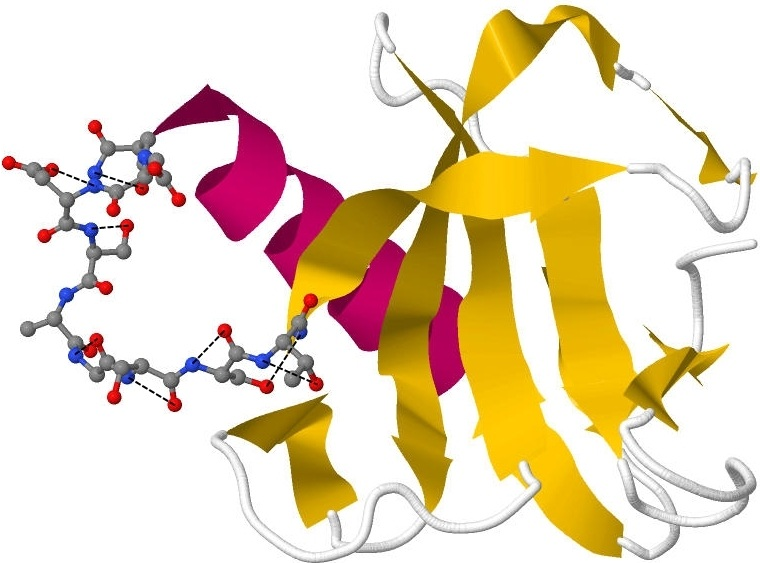
**The optimal decoy selected by our triplet potential for loop **1fus**(28:38)**. The decoy makes internal hydrogen bonds (black dashed lines) but few contacts with the protein frame.

For the test case of 1fus(28:38) loop target, Rosetta and the triplet scoring functions select, as best scored, decoys with RMSD > 3A from the native structure, albeit the other scoring functions as well as POC can correctly identify the decoy with best resolution. The decoy selected by triplet has a better scoring torsion angle combination, allowing for some favorable near residue neighbor interactions depicted as black dashed lines in Figure 15. These are either local backbone to backbone or side-chain to backbone hydrogen bonds. Our triplet scoring function evaluates a loop only by its internal local interactions. The cause this decoy is highly deviated from the native loop structure is that it makes few tertiary contacts with the rest of the protein, being rather detached from it (as shown Figure 15), it is solvated and unfolded, and therefore, cannot be stable. This is the reason for which the triplet scoring function should be used in conjunction with other distance-based potentials, a task that is accomplished here by our consensus POC method, which performs well for this target despite the triplet and Rosetta failures.

On the other hand, Rosetta's best scored decoy has the opposite problem: It makes some good contacts with the protein frame but has a poor choice of backbone torsion angle combinations. For example, the Thr37 residue has the following backbone torsion angle combination: phi = 80°, psi = -45°, which falls on a region of the Threonine's Ramachandran map that is disallowed due to local steric clashes. The success of the POC method in this case is justified by selectively relying on the other scoring functions that have good performances.

A somewhat opposite example is provided by the 1aac(16:20) target, where only the triplet scoring function selects decoys close to the native structure. All the other scoring functions select decoys with inferior torsion angle combinations. It seems that the distance-based scoring functions cannot accurately evaluate the local backbone interactions that are well described by our triplet torsion angle scoring function. Despite scoring a loop by its internal interactions only, our triplet scoring function proves itself as a valuable tool in the POC scheme. Our POC method heavily relies on the triplet scoring function to identify the near-native conformation in this case.

**Figure 16:**
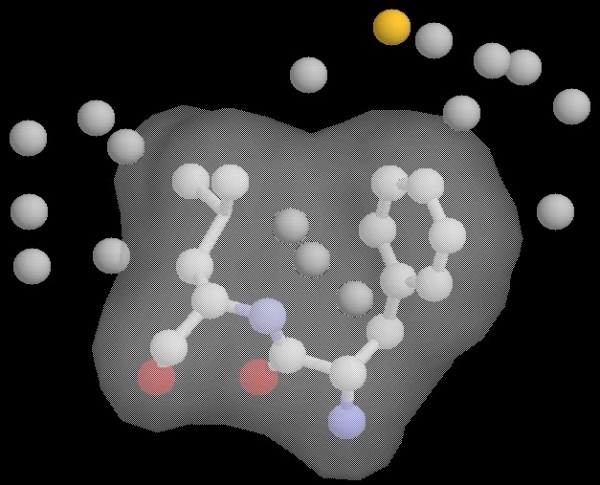
**Analysis of the native loop **1hbq**(31:38)**. The hydrophobic residues Phe36-Leu37 (enclosed by the surface) are buried in a stable protein hydrophobic core, being surrounded by many carbon atoms.

The only case, from all the 502 loop targets studied here, where POC fails to capture a native-like structure (within 0.5A cutoff) on the Parento Optimal Front, is the 1hbq(31:38) loop. This also means that none of the individual scoring functions can correctly identify a decoy with native-like conformation as the top-ranked one. 1hbq(31:38) is a special case where the native loop energy is a compromise between a poor loop internal energy and a very favorable energy of interaction with the rest of the protein. The loop internal energy is compromised by the Leu37 residue with an unfavorable backbone conformation (phi = 54°, psi = 93°) that scores poorly in every potential function. On the other hand, this residue participates, together with its loop neighbor Phe36, in a network of favorable hydrophobic interactions involving many atoms from the protein frame. In Figure 16, the two loop residues are shown enclosed by their external surface, together with the protein frame atoms that are closest to their side-chains. With the exception of a sulfur atom, all the other surrounding atoms are hydrophobic carbons. The two loop side-chains are hydrophobic themselves and form a hydrophobic core that is very favorable for the protein stability.

**Figure 17:**
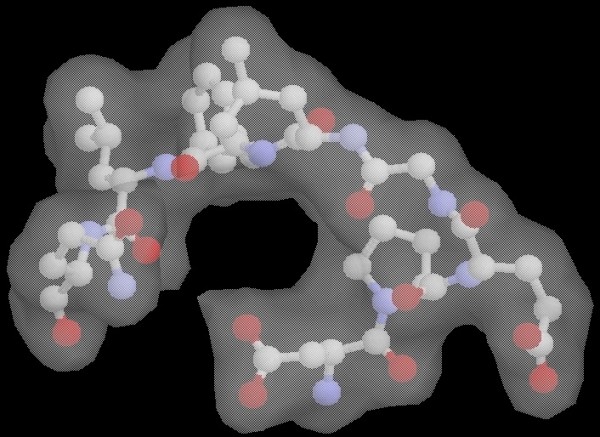
**The best decoy selected by POC for target **1hbq**(31:38)**. The decoy forms an unfavorable internal cavity that is not occupied by other protein atoms.

The best decoy selected by POC for this loop shows many favorable contacts, including the hydrophobic interaction between Phe36 and Leu37 side-chains. But they are not buried in a protein hydrophobic core in this case. Also, this decoy's surrounding surface, shown in Figure 17, forms a central internal cavity that is not filled with other protein atoms and is energetically unfavorable for this reason. None of the scoring functions is able to capture these important protein features, involving hydrophobic cores and internal cavities, because they are based on multiple-body cooperative interactions. As a result, our POC method is misguided in constructing the Pareto optimal front.

## Limitations of the POC Method

Similar to the other consensus methods, a limitation of the POC method depends on the accuracy of the scoring functions involved in the consensus scheme. If the large majority of the scoring functions have poor accuracy, the consensus scheme is unlikely to select decoys with high resolution. The effectiveness of the POC method also depends on the quality of the decoys generated. POC is a selection and ranking scheme and thus it is unable to generate better decoys than the best one in a decoy set.

Another minor disadvantage of the POC method is the decoy selection and ranking time when the decoy set is large. For a set of *N *decoys, the Pareto-optimal decoys selection and ranking time scaling is *O*(*N*^2^) because of the requirement of evaluating pair-wise decoy dominance relationship, whereas the ranking time scaling in regression-based, rank-based, or vote-based consensus methods is *O*(*N*). However, compared to the training time in regression-based method and the evaluation time for the scoring functions, the decoy selection and ranking time in the POC method is still rather small for a reasonable size of the decoy set.

## Conclusions

The POC method is shown to be effective in distinguishing the best models from the other ones within Jacobson's loop decoy sets and the membrane protein loop decoy sets. It is clear that a combination of multiple, carefully-selected physics- and knowledge-based scoring functions can significantly reduce the number of false positives compared to using an individual scoring function only. Moreover, identifying the decoys at the Pareto optimal front and ranking these decoys based on the fuzzy dominance relationship against the other decoys in the set have led to higher model selection accuracy in the POC method than in the other consensus strategies including rank-by-vote, rank-by-number, rank-by-rank, and regression-based methods. In addition to protein loop structure prediction, the POC approach may also be used in applications of protein folding, protein-protein interaction, and protein-ligand docking.

Our current POC implementation does not bias to any individual scoring function. However, there may still be improvement space for the POC method. For example, the POC may couple with a training algorithm to measure the efficiency of a scoring function and then certain bias to some scoring functions can be incorporated in evaluating the fuzzy Pareto dominance relation. This will be one of our future research directions.

## Authors' contributions

YL conceived and implemented the method and carried out the computation. IR performed the biological analysis. SC designed the computational experiment using physics-based energy function. EJ coordinated the study. YL, IR, SC, and EJ performed the result analysis. All authors read and approved the final manuscript.

## Supplementary Material

Additional file 1 **Efficiency of POC in Identifying the Native Structures**. The supplementary file describes the efficiency of the POC method with the native structure mixed in the decoy sets generated by Jacobson et al. The POC method also leads to less false positives compared to individual scoring functions. Click here for file
