# Supplementary material for: Improving predicted protein loop structure ranking using a Pareto-optimality consensus method
Source: BMC Struct Biol. 2010 Jul 20;10:22. doi: 10.1186/1472-6807-10-22 (PMC2914074; doi:10.1186/1472-6807-10-22)
Supplement: Additional file 1 — Efficiency of POC in Identifying the Native Structures. The supplementary file describes the efficiency of the POC method with the native structure mixed in the decoy sets generated by Jacobson et al. The POC method also leads to less false positives compared to individual scoring functions. [file 1472-6807-10-22-S1.DOCX]

### Efficiency of POC in Identifying the Native Structures

We applied the POC method with the native structure mixed in the decoy sets generated by Jacobson et al. To ensure that there is only one instance of native structure in the decoy set, we eliminate the OPLS-AA/SGB optimized native (MODEL 1) as well as the side-chain optimized native (MODEL 2) present in the original decoy sets. Moreover, due to the fact that the native structures are obtained from crystallized conformations, which usually have high energy values in a physics-based energy function, we excluded OPLS-AA/SGB from the scoring functions set.

Figure 1 shows the overall number of incorrectness in identifying the native structure in the 502 loop targets. One can find that the POC method, by integrating multiple scoring functions, has ~23% less false positive cases than the best individual scoring function in both identifying the native as the top ranked model and in placing the native among the top five ranked models. Figure 2 shows the success rate of identifying the native structure as the top model in 4-residue to 12-residue loop targets. Compared to other individual scoring functions, the triplet backbone dihedral scoring function has a higher success rate in short loop targets (4- and 5-residue) while Rosetta performs better in medium and long loop targets (6-residue and longer). In contrast, the POC selection method is superior or at least as good as the best individual scoring function for each target category. It is also interesting to notice that the POC selection method yields higher successful native structure identification rates in long loops than in short loops. This is due to the fact that the short loops have significantly smaller conformation space than the long loops and there are numerous models very close to the native structure in the decoy sets of most targets, and it is relatively difficult to differentiate the native structure from these many native-similar models. On the other hand, the large conformation space makes production of models close to the native structure difficult. In a number of long loop targets, there are no models within 1A RMSD from the native in the decoy set. Therefore, the POC method yields a higher native structure identification rate in the long loop targets.


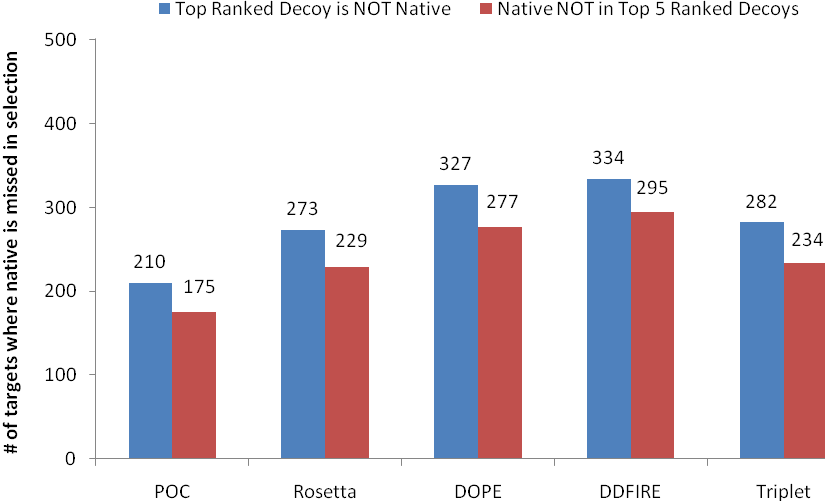


Figure 1: Number of cases where the top-ranked decoy is not a native and the native is missed in the top-5-ranked decoys in 502 loops of Jacobson’s decoy sets using the POC method and individual scoring functions. For each loop target, the native structure is mixed in the decoy sets


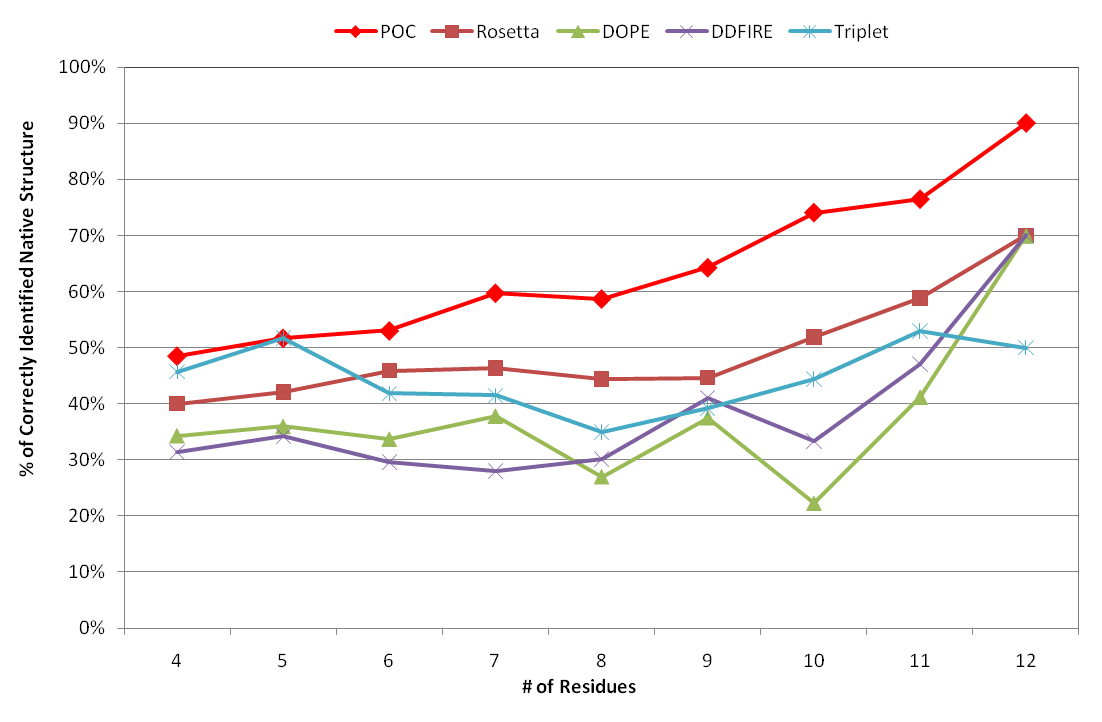


Figure 2: Percentage of correctly identifying the native structure when the native structure is mixed in Jacobson’s decoy sets in loop targets with different lengths

Figure 3 shows the percentage of targets where the native conformations are correctly identified in the membrane protein decoy sets. POC also exhibits the highest identification accuracy for short, medium, and long loops.


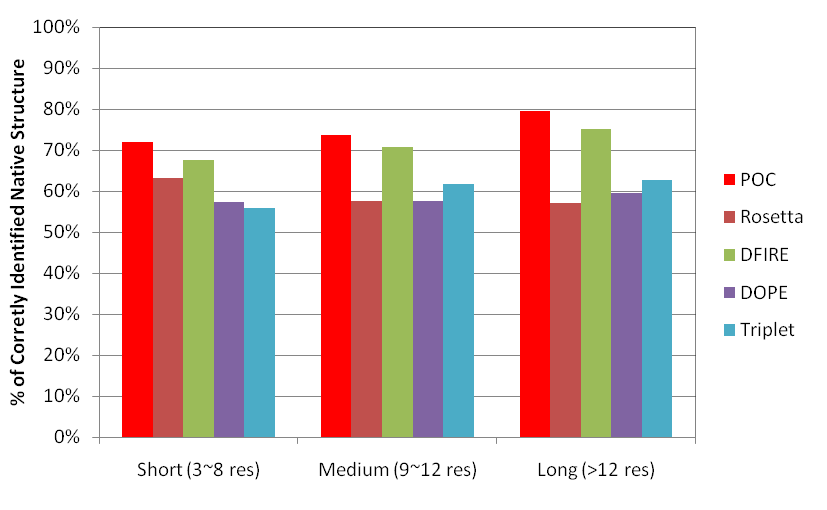


Figure 3: Percentage of correctly identifying the native conformation in the membrane proteins loop decoy sets
